# Challenges in Responding to the Ebola Epidemic — Four Rural Counties, Liberia, August–November 2014

**Published:** 2014-12-19

**Authors:** Aimee Summers, Tolbert G. Nyenswah, Joel M. Montgomery, John Neatherlin, Jordan W. Tappero

**Affiliations:** 1 Epidemic Intelligence Service; 2 Division of Global Health Protection, Center for Global Health, CDC; 3 Liberian Ministry of Health and Social Welfare; 4 CDC Kenya, Center for Global Health, CDC

The first cases of Ebola virus disease (Ebola) in West Africa were identified in Guinea on March 22, 2014 (1,2). On March 30, the first Liberian case was identified in Foya Town, Lofa County, near the Guinean border (3). Because the majority of early cases occurred in Lofa and Montserrado counties, resources were concentrated in these counties during the first several months of the response, and these counties have seen signs of successful disease control (4,5). By October 2014, the epidemic had reached all 15 counties of Liberia (6). During August 27–September 10, 2014, CDC in collaboration with the Liberian Ministry of Health and Social Welfare assessed county Ebola response plans in four rural counties (Grand Cape Mount, Grand Bassa, Rivercess, and Sinoe [Figure 1]), to identify county-specific challenges in executing their Ebola response plans, and to provide recommendations and training to enhance control efforts. Assessments were conducted through interviews with county health teams and health care providers and visits to health care facilities. At the time of assessment, county health teams reported lacking adequate training in core Ebola response strategies and reported facing many challenges because of poor transportation and communication networks. Development of communication and transportation network strategies for communities with limited access to roads and limited means of communication in addition to adequate training in Ebola response strategies is critical for successful management of Ebola in remote areas.

## Inadequate Training and Supplies

At the time of assessment, a total of 25 suspected, 16 probable, and 19 confirmed cases had been reported by the four counties: Grand Cape Mount (two suspected, four probable, and four confirmed), Grand Bassa (21 suspected, 12 probable, and 13 confirmed), Rivercess (one confirmed), Sinoe (two suspected and one confirmed) (7,8). Response teams in the four counties reported lacking adequate training in case investigation, contact tracing, infection control (including safe burial practices), and health education. Only Grand Bassa reported having teams trained in case investigation and contact tracing at the time of its first reported case. County health officials in Rivercess, Sinoe, and Grand Cape Mount reported that corpses had been transported by persons without prior training in safe burial practices and health care workers had not received any training in transporting a patient with possible Ebola. Grand Bassa and Grand Cape Mount health officials reported having a functioning ambulance, whereas the other two counties reported no functioning ambulance. Only Grand Bassa health officials reported having an ambulance crew trained in loading and transporting a suspected Ebola patient.

**FIGURE 2:**
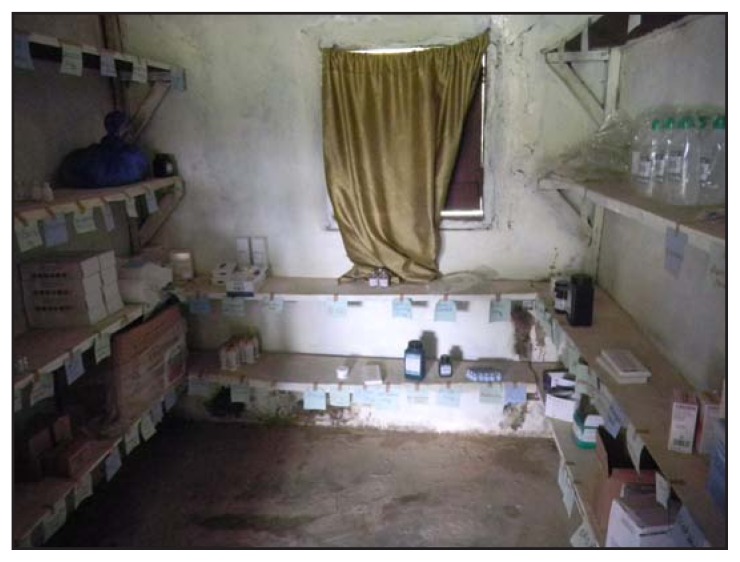
Mostly empty shelves in a clinic lacking essential drugs in Gblorseo Town — Rivercess County, Liberia, September 2014

Only one laboratory technician had been trained to safely collect and handle specimens from a possible Ebola patient in Grand Cape Mount and Grand Bassa, whereas Sinoe health officials reported having no laboratory technicians trained in handling Ebola specimens. In all four counties, health care workers had a limited supply of personal protective equipment, but had not received training in its proper use. Essential drugs were reported lacking at rural health clinics for all four counties (Figure 2).

## Poor Transportation and Communication Networks

**FIGURE 3:**
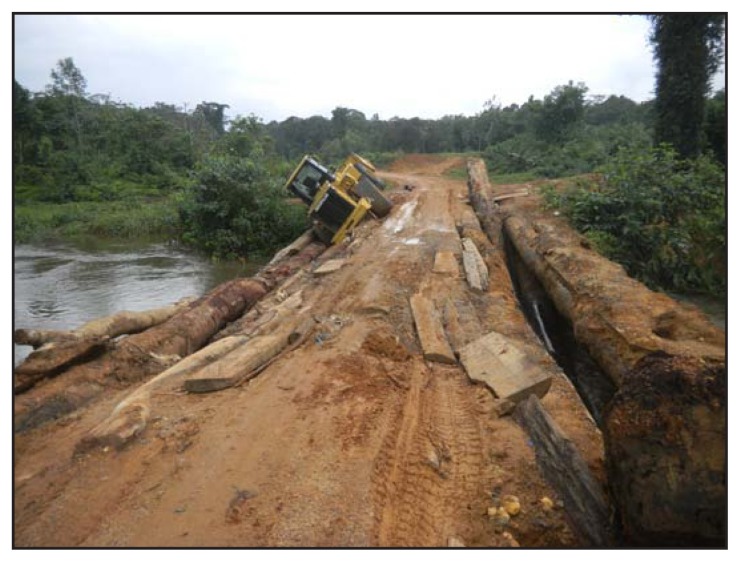
A nearly impassable bridge on the road connecting Sinoe County with Monrovia, the closest location with Ebola treatment units — Liberia, September 2014

Case investigation teams in Grand Bassa and Sinoe reported walking for up to 8 hours from the nearest road and crossing several rivers to reach communities where cases had been reported and where contact tracing and safe burials had not occurred because there were no trained personnel. During the rainy season (July–December), county health officials in all four counties reported existing roads often were impassable or could only be used by four-wheel drive vehicles, which were rarely available, making it impossible to transport laboratory specimens or patients from these counties to Ebola treatment units located in Monrovia (Figure 3). Many communities in these counties reported a lack of telephone coverage, making it difficult for community leaders to notify county health teams about suspected Ebola patients, to arrange a clinical evaluation, or to receive laboratory test results in a timely manner. Because of poor connectivity, workers in Rivercess County reported driving 6 hours round-trip to the next county to send surveillance reports to the Ministry of Health and Social Welfare over the Internet. County health officials in Sinoe reported a 3-day lag in receiving laboratory test results.

As of November 21, 100 suspected, 114 probable, and 101 confirmed Ebola cases were reported from the four counties: Grand Cape Mount (38 suspected, 26 probable, and 32 confirmed), Grand Bassa (42 suspected, 68 probable, and 33 confirmed), Rivercess (nine suspected, 17 probable, and 18 confirmed), Sinoe (11 suspected, three probable, and 18 confirmed) (9). Although additional training in case investigation, contact tracing, infection control, safe burials, and health education had reportedly occurred in all four counties during late September–November, these counties still reported facing many of the same challenges identified in the August 27–September 10 assessments, and case counts continued to increase.

## Continuing Challenges

Continuing challenges as of November, included lack of trained personnel in remote areas and logistic constraints regarding travel and communication. Grand Cape Mount officials reported a continued lack of vehicles to transport patients and an insufficient number of trained contact tracers to manage the growing number of cases. In Grand Bassa, health officials reported an ongoing lack of ambulances and communication between the Ebola treatment units and county health teams regarding patient status and laboratory results. In addition, the capacity to investigate cases in remote areas in Grand Bassa was reportedly insufficient because of limited trained personnel and transportation capabilities. In Sinoe, contact tracing and supervision was reportedly lacking in remote areas, and poor road conditions and vehicle maintenance continued to make transportation challenging for patients and county health teams. Rivercess had incorporated active case finding into its response strategy but difficulties with transportation and communication networks reportedly remained.

The Ebola epidemic in Liberia presents unique challenges not only from its spread into crowded urban environments (10) but also its occurrence in remote communities. As in urban counties, county and district health teams in rural counties with remote regions need adequate training in 1) case reporting; 2) case investigation; 3) case management; 4) contact tracing; 5) safe burials; 6) safe collection, processing, and transport of blood specimens for testing; and 7) development of a county-level incident management system. However, in rural counties, few roads, poor road conditions, and an overall lack of vehicles, vehicle maintenance, Internet connectivity, and limited telephone network coverage impedes epidemic control. Development of innovative communication and transportation network strategies for communities with limited access to roads and limited means of communication is critical for successful management of Ebola in remote areas. These strategies are needed to ensure essential supplies such as personal protective equipment, chlorine/disinfectants, body bags, and sprayers can reach county health teams and suspected, probable, and confirmed Ebola patients can be transported and isolated in Ebola treatment units as soon as they are identified (10).

## Figures and Tables

**FIGURE 1:**
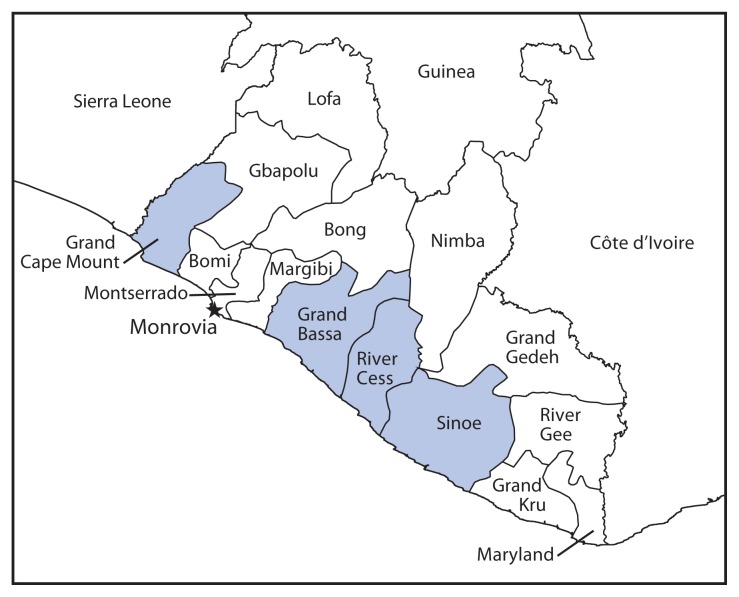
Location of the four rural counties assessed for challenges associated with Ebola epidemic response plans — Liberia, August–November 2014
